# Landmarks in Gynecology

**Published:** 1894-11

**Authors:** 


### Book Reviews.

---

Landmarks in Gynecology. By Byron Robinson, B. A., M. D., Chicago, Ill., Professor of Gynecology in the Chicago Post-Graduate School; Gynecologist to the Woman's Hospital, the Charity Hospital and the Post-Graduate Hospital. Vols. I. and II. Geo. S. Davis, Detroit, Mich. 1894.

Byron Robinson is a familiar name to every gynecologist. As a teacher, a writer and a gynecologist, he stands at the top. Those who admire him will not be disappointed in his "Landmarks in Gynecology." Those concise little volumes are written in the most lucid, forceful style and thoroughly cover the subjects under discussion, having not a superfluous paragraph or word.

For the purpose of impressing the chief features in gynecology, he has divided into six prominent landmarks the diseases from which the patient most probably suffers—Namely: Anatomy; Menstruation; Labor; Abortion; Gonorrhœa; Tumor. The chapters on Gonorrhœa in Women will fully reward anyone for reading the book. The material upon which these lectures are founded has been gleaned from a large personal experience in gynecological operations, in dissections, and in post-mortems.

We take great pleasure in highly endorsing this book. It is instructive from beginning to end, practical throughout, and original to a marked degree.

